# Striking the balance: Configurations of causation and effectuation principles for SME performance

**DOI:** 10.1371/journal.pone.0302700

**Published:** 2024-06-28

**Authors:** Xiaoyu Yu, Wenjing Zhao, Xinchun Wang, Xiaoshu Ma, Gang Cao

**Affiliations:** 1 School of Management, Shanghai University, Shanghai, China; 2 John Chambers College of Business and Economics, West Virginia University, Morgantown, WV, United States of America; Industrial University of Ho Chi Minh City, VIET NAM

## Abstract

Causation and effectuation are two fundamental decision-making logics that managers use for crucial firm strategic decisions. However, existing research has yet to agree on the relationship between the two logics, supporting both the substitution and complementarity of causation and effectuation in influencing firm performance. This leaves us with a puzzle: How do causation and effectuation combine in balance to improve firm performance? To address the gap, we utilize a fuzzy set qualitative comparative analysis (fsQCA) with data collected from 344 small to medium-sized enterprises (SMEs) in China to uncover the dynamic relationships between the two logics. Our findings indicate that causation or effectuation alone is insufficient to achieve superior firm performance. By distinguishing between four dimensions of effectuation, we identify three types of configurations for high performance: (1) causation with promotion-focused effectuation principles; (2) causation with prevention-focused effectuation principles; (3) causation with hybrid-focused effectuation principles. More importantly, we find that the effectiveness of the configurations depends on the firm development stage. Our findings provide SMEs with practical insights into how to effectively choose their decision-making logic when faced with different firm growth challenges.

## 1. Introduction

In entrepreneurship, making critical decisions hinges on a delicate balance between goal-driven “causation” and action-oriented “effectuation” strategies. Both researchers and practitioners have long acknowledged these two main decision-making logics as pivotal in shaping firm performance [1–3]. However, the findings on the relationship between causation, effectuation, and firm performance remain inconclusive [2, 4–10]: some argue that causation and effectuation are substitutes [11, 12], while others suggest that they complement each other to jointly improve firm performance [2, 5, 6, 10].

To provide more insights, recent research has started to use a configurable approach to explore the complex relationship between causation and effectuation. However, previous studies often view effectuation as a unidimensional construct [13]. This limits our understanding of the true relationship between causation and effectuation because effectuation has four different dimensions with each reflecting different or even opposing cognitive processes. To address these gaps, this study intends to identify configurations of causation and effectuation principles that help improve firm performance using a holistic approach. Specifically, we aim to investigate the following research question: “*How do causation and effectuation principles combine configurationally to achieve high firm performance*?”

To answer this question, we use a fuzzy set qualitative comparative analysis (fsQCA) to explore configurations of causation and effectuation principles that are associated with high firm performances. Compared to conventional correlation-based methods, fsQCA relies on causal conjunctions and posits that outcomes stem from the interaction of multiple conditions. By doing so, it identifies all theoretically possible configurations and assesses their necessity and sufficiency for the observed outcome [14]. By analyzing a sample of 344 software SMEs, our findings show that causation or effectuation alone is insufficient to achieve high performance. Instead, we uncover three configurations of decision-making strategies: (1) causation with promotion-focused effectuation principles, (2) causation with prevention-focused effectuation principles, and (3) causation with hybrid-focused effectuation principles. As firms likely present different needs for strategies under different stages of their lifespan, we also find that the effectiveness of these configurations varies across a firm’s development stage.

The study makes two important contributions to the literature. First, we contribute to the ongoing debate on the relationship between causation and effectuation [2, 4–10]. While managers often rely on these two decision-making logics for important strategic decision-making, insights are not clear as to how the two logics interplay with each other. We add new insights by distinguishing the different effects of each effectuation dimension and uncovering the dynamic relationships between the two logics. Second, our study identifies three configurations of causation and effectuation and highlights their different roles during firms’ different development stages. These findings provide practical insights for managers when making important decisions when faced with different situations.

## 2. Literature review

### 2.1 Effectuation theory

Building on the “bounded rationality theory” and the “opportunity creation view,” effectuation theory challenges the dominance of causation that emphasizes goal-oriented rational analysis and formal planning in management and entrepreneurship literature and proposes effectuation as a new decision-making logic [1]. Sarasvathy (2001, p. 245) defines effectuation and causation as “processes take a set of means as given and focus on selecting between possible effects that can be created with that set of means” and “processes take a particular effect as given and focus on selecting between means to create that effect,” respectively [1]. This study views causation and effectuation as strategic decision-making logic for implementing firm strategy [9, 15, 16].

#### 2.1.1 Causation

Causation, a dominant logic in entrepreneurship literature [1, 15], is theoretically driven by neoclassical rational decision-making approaches [17]. Based on the assumption that the future is predictable, causation relies on analyzing market trends to understand competition in the market and select possible means to realize predetermined goals [12]. This decision-making approach entails systematic searching for opportunities by relying on marketing and business plans [17]. The central question in causation logic is what resources are needed to attain the selected goals [1, 12, 15, 17].

#### 2.1.2 Effectuation

Effectuation assumes that the future is unpredictable and emphasizes transforming a current set of means into convergent new goals and co-creating new opportunities [6]. As the literature suggests, effectuation consists of four dimensions: experimentation, flexibility, affordable loss, and pre-commitments [17–19].

The experimentation principle is defined as a series of trial-and-error changes along the various principles of a strategy over a relatively short period to create something new with the resources at hand [17]. The effectuation process reflects a series of experiments with business models that allow firms to formulate and crystallize strategic goals in an unpredictable future. The flexibility principle emphasizes confronting a contingency rather than trying to avoid it and turning it into a profitable opportunity [17]. The flexibility principle facilitates firms’ responses to environmental changes to quickly take advantage of contingencies without restrictions under uncertainty. The affordable loss principle emphasizes the importance of considering the potential risks and losses associated with investments, suggesting that firms should make decisions within the bounds of acceptable loss [9, 17]. The pre-commitments principle focuses on constructing cooperative relations and independent stakeholders to establish strategic alliances instead of systematic competition analysis [17]. For instance, predefined agreements with stakeholders in innovation activities can encompass technical assistance, innovation output, and financial support commitments.

In the extant literature, most scholars have focused on the overall effect of effectuation on strategic decision-making. However, recent studies have argued that various effectuation principles may reflect different or opposing cognitive processes [13, 19]. For example, Palmié et al. (2019) focus on decision-making logic motivations and strategic dispositions and distinguish between promotion-focused and prevention-focused effectuation principles [19–21]. Experimentation and flexibility, preferring to pursue success rather than avoiding mistakes, embody the promotion focus [19–21]. Pre-commitments and affordable loss pursue avoidance-oriented or “minimal” goals, reflecting the prevention focus [19–21]. Similarly, recent studies have suggested that different effectuation principles have varying effects on various performance outcomes [22–28].

### 2.2 Causation, effectuation, and firm performance

Previous research examining the impact of causation and effectuation on firm performance has taken different perspectives on how these decision logics interact (see S1 Table). For example, one stream of research interprets causation and effectuation as two substitutable decision logics, each leading to high performance. For instance, Brettel et al. (2012) view causation and effectuation as two sides of a continuum, forcing decision-makers to choose between two options in each of four dimensions: means or goals, affordable loss or expected returns, partnerships or competitive market analysis, and acknowledging or overcoming the unexpected [11–12, 17]. This literature stream also believes that certain boundary conditions influence the balance between causation and effectuation. For example, effectuation is more effective for start-ups that usually face high levels of environmental uncertainty [2, 5]. At the same time, established firms are more likely to grow through causation [29].

Contrary to the literature mentioned above, another stream of research argues that the two decision-making logics are complementary [30, 31]. As Sarasvathy (2001, p.245) states, “Both decision-making logics are integral parts of human reasoning and can occur simultaneously, overlapping and intertwining over different contexts of decisions and actions” [1]. Studies have proved that causation and effectuation can occur alternatively or simultaneously. For example, Berends et al. (2014) analyzed 352 events in five small manufacturing firms’ product innovation processes and found that causation and effectuation were applied [5]. Other studies have explored the interaction between causation and effectuation and found that the combined use of the two decision-making logics has a more significant impact on venture performance than causation or effectuation alone [2, 6, 7, 10, 32, 33]. Studies advocating complementarity between causation and effectuation have recognized firm development stages as critical contextual factors [1, 2, 5, 30, 34]. For example, Sitoh et al. (2014) suggest that decision-making mechanisms should be configured in specific ways during different phases of new product creation [35].

Upon reviewing the literature, it is evident that previous studies predominantly relied on traditional regression methods to explore the relationships among causation, effectuation, and firm performance [26, 36–40]. However, the relationships among causation, effectuation, and firm performance are inconsistent. Despite the advantages of regression analysis, this method assumes independence and non-interaction among variables. However, intricate interrelationships exist between these two decision-making logics. Recent research has focused on the configuration perspective concerning causation and effectuation for achieving high firm performance [30]. We aim to extend the prior literature by providing insights into how causation and the four dimensions of effectuation (including experimentation, flexibility, affordable loss, and pre-commitment) balance from a configurational perspective and by revealing the successful logical configurations for high firm performance.

## 3. Research design

### 3.1 fsQCA

This study aims to explore high-performance configurations of causation and effectuation principles in different contexts. To achieve this goal, we use a methodological approach to capture intricate causal relationships between interconnected elements [41]. FsQCA has gained increasing recognition as a viable method for examining complex phenomena in strategic management and entrepreneurship [41–44] and serves the purpose of this study very well.

First, fsQCA lies in its utilization of causal conjunction and posits that outcomes stem from the interaction of multiple conditions. This provides a distinct advantage compared to conventional correlation-based methods [41]. For instance, analyzing configurations using regression models would entail modeling higher-order interactions, posing computational limitations and challenges in interpretation [42]. In contrast, fsQCA identifies all theoretically possible configurations and assesses their necessity and sufficiency for the observed outcome [14]. Additionally, it can identify the most critical elements in each relevant solution [42].

**Fig 1:**
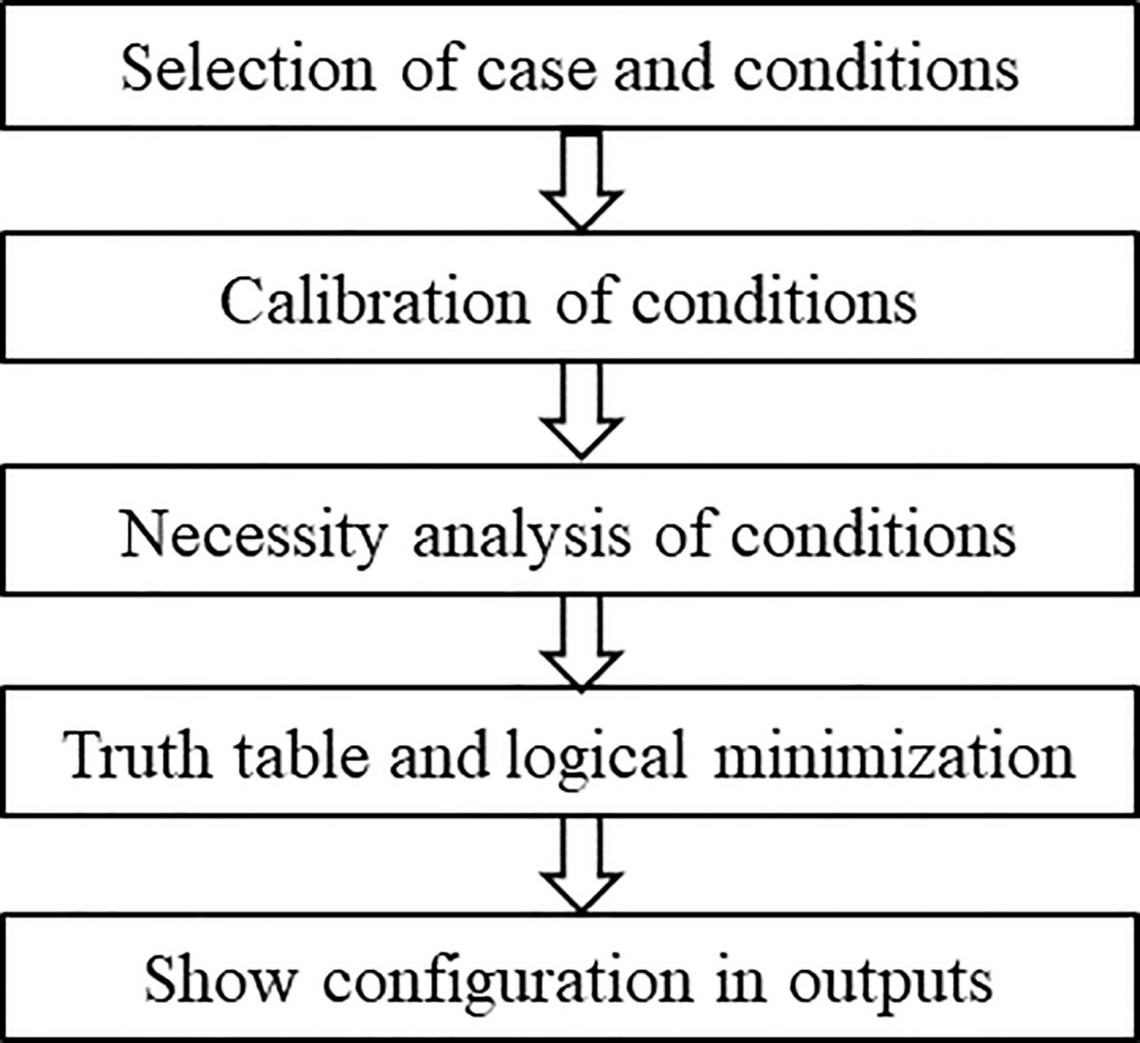
fsQCA data analysis process.

Furthermore, fsQCA provides another advantage by accounting for equifinality [43]. Equifinality, which assumes multiple pathways leading to the same outcome, enables researchers to assess the number and complexity of different routes to a desired outcome [43]. While regression techniques focus on the isolated effects of individual variables, fsQCA offers insights into both first-order equifinality, where configurations differ in their core conditions, and second-order equifinality, where configurations share the same core conditions but differ in peripheral conditions [42]. Specifically, we compared regression analysis and fsQCA methods, as described in S2 Table. Given our aim to uncover multiple high-performance configurations involving causation and effectuation principles and our interest in causal conjunction and equifinality, the fsQCA approach aligns well with our research objectives. The analysis steps of the fsQCA method are shown in Fig 1.

### 3.2 Data collection

We consider Chinese SMEs operating in the software industry. The software industry is characterized by rapidly changing technologies, competitive markets, and customer needs. As software firms grow, the customers they face become more heterogeneous, and the characteristics of the software change considerably (from highly standardized to personalized for customers), which requires a flexible combination of decision-making logic. Therefore, this industry is theoretically relevant for studying decision-making logic under conditions of uncertainty [9, 45–47]. We choose SMEs instead of large firms based on two considerations: First, globally, SMEs are considered vital to economies, contributing to >90% of all companies in many countries [48, 49]. In China, SMEs represent 98% of the country’s companies. Second, the organizational structure of SMEs is typically flatter and more straightforward than larger firms. This simplicity often lends SMEs greater flexibility in their decision-making processes [50]. Additionally, such an organizational structure facilitates more direct observation and analysis of the impact of decision-making logic. Therefore, we believe that focusing on software SMEs is representative.

We analyzed the software industrial parks in four cities (Beijing, Shanghai, Hangzhou, and Changchun) to identify these software SMEs. Based on the firm directories of software industrial parks in various regions, we randomly selected 800 SMEs with less than 500 employees. We perused the firms’ web pages and made confirmatory phone calls to identify senior managers and verify that the respective firms met our inclusion criteria. Before conducting the study, we informed all participants of the research purpose and guaranteed the confidentiality of all responses. We obtained voluntary informed consent signed by the participants. Our research has been exempted from ethical review by the Ethics Committee of Shanghai University.

Effective academic procedures for questionnaire design require the development of a fully standardized questionnaire that can be used to collect relevant data [51]. First, we developed the questionnaire in English based on previous studies [9, 17]. Then, we translated the questionnaire back with the help of two entrepreneurship researchers fluent in English and Chinese. Before the formal investigation, we invited ten software firms to complete our questionnaire as a pilot test. Based on their feedback, we modified the questionnaire to enhance its clarity. Subsequently, we distributed questionnaires to the chief executive officers (CEOs) or other senior managers through WeChat, email, or on-site. The survey database contained 344 questionnaires completed between June 20, 2021, and October 30, 2021. Firm’s decision-making logic is fundamentally stable and does not undergo significant changes due to short-term environmental fluctuations. Table 1 summarizes the main sample characteristics.

**Table 1:** Sample descriptive statistics.

Characteristic		Frequency	Percentage
Gender	Male	238	69.1
	Female	106	30.9
Age	29 years or younger	154	44.8
	30–40 years	169	49.1
	41–50 years	20	5.8
	51 years or older	1	0.3
Education	Lower than bachelor	26	7.6
	Bachelor	236	68.6
	Master	78	22.7
	Ph.D.	4	1.2
Ownership	Private firm	226	65.7
	Non-private firm	118	34.3
Firm age	1–8 years	166	48.3
	9 years or above	178	51.7
Firm size	1–100 employees	101	29.4
	101–200 employees	93	27.0
	201–300 employees	72	20.9
	301–500 employees	78	22.7
Location	Beijing	115	33.4
	Shanghai	113	32.8
	Hangzhou	59	17.2
	Changchun	57	16.6

### 3.3 Measurement

#### 3.3.1 Causation and effectuation principles

Following Chandler et al. (2011), we measured causation and effectuation principles using a 5-point Likert scale [17]. Specifically, causation consisted of seven items (Cronbach’s α = 0.87); experimentation was measured by three items (Cronbach’s α = 0.60); flexibility consisted of four items (Cronbach’s α = 0.75); affordable loss contained three items (Cronbach’s α = 0.78); and pre-commitments were measured using six items (Cronbach’s α = 0.80).

#### 3.3.2 Firm performance

According to Dess and Robinson (1984), unlisted firms usually do not have objective measures of firm performance. They are significantly associated with subjective measures of firm performance on a 7-point Likert scale [52]. Thus, we used subjective items reported by the respondents to measure firm performance (Cronbach’s α = 0.83). The respondents were asked to answer how they would rate their own firm’s performance relative to other software firms in the same city, in the same market niche, and in the same industry [9].

#### 3.3.3 Start-ups and established firms

To explore the configurations of decision-making logics at different firm development stages, we divided the sample into two subsamples based on firm age. Based on Zahra and Bogner (2000), we classified firms within 8 years of establishment as start-ups and firms over 8 years old as established firms [45]. Our two subsamples consisted of 166 start-ups and 178 established firms, respectively.

The specific items for each variable are shown in S3 Table.

## 4. Fuzzy-set qualitative comparative analysis

### 4.1 Conditions calibration

The first important step in performing fuzzy-set analysis is to convert the raw data into set data, a process known as calibration [53–55]. Calibration requires setting three anchor points: full membership, full non-membership, and maximum ambiguity. We used direct calibration to convert the raw data obtained on interval scales into fuzzy-set scores ranging from 0 to 1 [56], with 0 implying full non-membership and 1 implying full membership. To set meaningful thresholds for any condition, it has been suggested that calibration anchors be based on valid theoretical criteria [57]. Referring to previous studies, we selected 95^th^–50^th^–5^th^ percentile calibration to set three anchor points [58]. After completing the above calibration, a constant of 0.001 was added to 0.5 in our study to avoid the lack of a theoretical basis for classifying attribution in cases with a fuzzy affiliation score of 0.5 [41]. The anchor points for each variable are shown in Table 2.

**Table 2:** Data calibration.

Conditions	Set membership			
	Variable	Fully in	Crossover point	Fully out
Causation	Causation	4.86	4.00	3.02
Effectuation	Experimentation	4.33	3.33	2.00
	Flexibility	4.75	4.00	3.25
	Affordable loss	4.95	4.00	2.38
	Pre-commitments	4.67	4.00	2.83
**Outcome**	Firm performance	6.33	5.33	3.67

### 4.2 Analysis of necessary conditions

After the calibration, we analyzed the necessary conditions to assess whether any of these five conditions could be essential to lead to the outcome. In the process of fsQCA, if the consistency score is higher than the threshold of 0.9 [54], a single condition is necessary for the outcome variable. As shown in Table 3, none of the conditions exceeded the threshold. The result means that no single factor had a dominant effect on firm performance; instead, the effect resulted from multiple conditions acting together.

**Table 3:** Analysis of necessary conditions.

Conditions	Outcome	
	High firm performance	~ High firm performance
causation	0.79	0.64
~ causation	0.52	0.73
experimentation	0.71	0.65
~ experimentation	0.56	0.67
flexibility	0.78	0.68
~ flexibility	0.51	0.66
affordable loss	0.66	0.64
~ affordable loss	0.65	0.73
pre-commitments	0.73	0.63
~ pre-commitments	0.57	0.73

Note: ~ means the absence of. For example: ~ causation = absence of high causation.

### 4.3 Construction of truth tables

After analyzing the necessary conditions, we identified the possible configurations of conditions by constructing truth tables. In constructing the truth table, two thresholds must be set: frequency and consistency. Frequency describes the number of cases (sample firms) that match a given configuration. The frequency threshold is generally based on the sample size [55, 56], and because our study involved 166 start-ups and 178 established firms, we set the case frequency threshold to 3. At this point, at least 75% of the firms with high firm performance were included in the analysis [55, 56]. Consistency is the degree to which a combination of conditions produces a particular outcome (e.g., high firm performance). Referring to previous studies, we set the consistency value to 0.8, above the threshold of 0.75 [42, 59]. In addition, we set the threshold for inconsistency (PRI) to 0.7, with PRI indicating the level of consistency after eliminating the configurations that occurred in both high- and low-performance firms [41].

The fsQCA analysis yielded three solutions with different levels of complexity: complex, intermediate, and parsimonious [59, 60]. This paper reports the intermediate solution, supplemented by the parsimonious solution, to distinguish the core and peripheral conditions [41]. As shown in Table 4, we concluded that there were five configurations of causation and effectuation principles to achieve high firm performance.

**Table 4:** Configurations of conditions for high firm performance.

	Start-ups		Established firms			
	*Causation and promotion- focused effectuation principles*		*Causation and promotion- focused effectuation principles*		*Causation and prevention- focused effectuation principles*	*Causation and hybrid- focused effectuation principles*
		*(S1)*	*(E1a)*	*(E1b)*	*(E2)*	*(E3)*
Causation	Causation	**●**	**●**	**●**	**●**	**●**
Effectuation	Experimentation	**●**	**●**		⊗	**●**
	Flexibility	**●**	**●**	**●**		
	Affordable loss			**⊗**	**●**	**●**
	Pre-commitments			**⊗**	**●**	**⊗**
	Consistency	0.860	0.840	0.893	0.894	0.882
	Raw coverage	0.564	0.590	0.397	0.364	0.381
	Unique coverage	0.564	0.135	0.020	0.013	0.045
Overall solution consistency		0.860	0.828			
Overall solution coverage		0.564	0.686			

Note: ● indicates the presence of a condition, while ⊗ indicates its absence. Large characters indicate core conditions, and small characters indicate peripheral conditions. Blanks indicate not caring about a condition

### 4.4 Research results

In the start-up stage, the consistency of the solution was 0.860, implying that 86% of all cases of start-ups satisfying the S1 configuration presented a high level of performance. In the maturity stage, the consistency of the solution was 0.828, indicating that 82.8% of all cases of established firms satisfying the E1a, E1b, E2, and E3 configurations showed a high level of performance.

As Palmié et al. (2019) suggest, experimentation and flexibility principles are dominated by a promotion focus concerning potential gains, while affordable loss and pre-commitments principles are dominated by a prevention focus concerning potential losses [19]. Following this approach, we identified each decision-making strategy configuration based on the core conditions contained and the characteristics of the decision-making logic.

#### 4.4.1 Configurations of conditions for high performance in start-ups

Solution S1, or *causation and promotion-focused effectuation principles*, consisted of causation, experimentation, and flexibility as the core conditions. Solution S1 indicates that firms achieve high performance by combining causation and promotion-focused effectuation principles without necessarily adopting other effectuation principles. This suggests that a complementary effect in causation and promotion-focused effectuation principles.

The biggest problem start-ups face is resource constraints. Causation emphasizes goal-setting, and the positive effects of goal-setting on firm performance have long been validated [61]. Moreover, their legitimacy will be enhanced when start-ups set specific goals and develop business plans. This is because the specificity in strategic goals and business plans communicates the feasibility and reliability of their business idea to investors, thereby increasing the chances of surviving the early stages of the life cycle [62]. However, causation also entails certain drawbacks, such as excessively rigid implementation plans that may result in firms needing more serendipitous opportunities.

Promotion-focused effectuation principles emphasize utilizing all incidental factors to seize new opportunities, which can effectively compensate for the shortcomings of causation. Specifically, experimentation encourages firms to experiment in different strategic directions, considering various alternatives [17, 38]. Trial-and-error strategies overcome barriers to obtaining more resources and opportunities, which can help start-ups establish a viable competitive basis [2, 63]. Valuable resources and competitive advantages improve firm performance [64]. Flexibility means that firms are sensitive enough to quickly adjust their strategic layouts according to changes in the market environment. For example, once there are signs of investment failure, firms can switch to new business opportunities, which helps start-ups adapt quickly to the external environment [6, 26, 65, 66]. Overall, the combination of causation and promotion-focused effectuation principles can increase start-ups’ chances of survival and help them capture and exploit more new opportunities for growth.

We contacted two firms with high performance for semi-structured interviews (see S4 Table). The interviews also supplemented the above argumentation. When asked what kind of decision-making logic companies typically adopt in the start-up phase (e.g., planning, trial and error, staying flexible, interacting with stakeholders, or setting acceptable losses), the CEO of Company A explained the following:

The company had just been established, and we had to figure out which industries and markets to target. We developed a detailed business plan to increase investment opportunities. However, that did not mean we rigidly executed those strategies. In executing our plan, we tried various approaches to understand which would be more effective for our strategy and goals. If we could not find what worked, we had to return t**o the drawing board and see where our plan went wrong.**

#### 4.4.2 Configurations of conditions for high performance in established firms

Solution E1a and E1b, or *causation and promotion-focused effectuation principles*. This suggests that there is also complementary value in causation and promotion-focused effectuation principles for established firms.

Established firms have extensive operational experience and are well-equipped to predict the future, as they are skilled in information about market conditions, competitor conditions, and product-related technologies. Using causation, firms select and ultimately achieve their revenue maximization goals [67]. However, established firms have a much higher level of institutionalization and standardization than start-ups, resulting in firms focusing on extracting profits from current products or services and being less likely to explore new opportunities [68]. Promotion-focused effectuation principles encourage established firms to experiment in different strategic directions, exploring new opportunities and using existing resources to create new value. Causation and promotion-focused effectuation principles help established firms overcome organizational inertia and achieve sustained growth [38, 39].

Compared to solution S1, solution E1a shows flexibility was no longer a core condition but had become peripheral. When asked how the decision-making logic changed in the later development stage compared with the start-up stage, the CEO of Company A added:

As the company grows, its ability to learn and flexibility gradually deteriorate. When the company entered a later development stage, we had to establish many formal rules to manage it better. The company gradually developed organizational inertia, and flexibility became less critical.

Further comparing E1a and E1b, we find that E1b needs to avoid prevention-focused effectuation principles while pursuing promotion-focused effectuation principles. It is reasonable because firms must allocate attention and time when pursuing different focus logics [69, 70]. The simultaneous use of promotion- and prevention-focused logic may result in contradictory outcomes: increased firm costs or reduced operational efficiency [9].

Solutions E2 can be labeled *causation and prevention-focused effectuation principles*. Solution E2 consisted of causation and pre-commitments as the core conditions and affordable loss, with the absence of experimentation as the peripheral conditions. This suggests a complementary relationship between causation and prevention-focused effectuation principles, but this appears to be the case only in firms that explicitly do not use the experiment principle. This approach aided established firms in setting goals that align with their acceptable level of risk and potential losses while utilizing pre-commitments from external stakeholders to mitigate uncertainty [12, 71].

Solution E3 can be labeled *causation and hybrid-focused effectuation principles*. Solution E3 consisted of causation and experimentation as the core conditions and affordable loss and the absence of pre-commitments as the peripheral conditions. The solution helped the established firms set goals and use trial and error within their affordable loss and risk. By doing so, the established firms could avoid higher resource losses and waste due to excessive risk-taking [38, 40, 72].

Further comparing E2 and E3, we found a partial trade-off between experimentation and pre-commitments. The firms that focused on experimentation were less dependent on partners, whereas those that attached importance to pre-commitments were less focused on experimentation. This finding further suggests that firms do not have to adopt a complete set of effectuation principles but can choose a specific combination of effectuation principles based on their circumstances. Experimentation involves trying new ideas, shifting work processes and steps, and being open and curious about how things happen and develop. Pre-commitments, however, emphasize pre-establishing collaborative relationships with relevant stakeholders. Such locked-in value networks constrain incumbent firms’ incentive for and likelihood to adopt disruptive technologies for innovation [73]. Using both principles simultaneously may lead to conflicting results, so firms must consider whether they should use experimentation or pre-commitments. The interviews verified our suspicions. When we asked whether pre-established partnerships would hinder innovation, the CEO of Company B responded as follows:

When we take a deposit from a client, we have to deliver strictly. Even if we do some “micro-innovations,” they can be puzzling and even offensive to the client. Therefore, we are more conservative in executing the contract and do not encourage our employees to jump out of the contract and try it on their own. We are not a small company anymore, and even if these attempts might benefit the client, it would still make us questionable to the client regarding our professionalism.

## 5. Discussion

As discussed in the literature review section, previous studies in entrepreneurship have primarily focused on analyzing causation and effectuation as separate concepts. While recent studies have started to explore their coexistence in entrepreneurial processes, our understanding of the interaction between two decision-making logics and their combined effect on firm performance still needs to be improved [74, 75]. Our study findings indicate successful configurations of causation and effectuation principles across firm development stages.

### 5.1 Conclusion

A key discovery of this research pertains to the central role of causation. Causation has consistently been a core ingredient in all solution terms. Prior studies indicate that causation is more relevant to established firms, as planning analysis is more effective in relatively stable environments. However, our research shows that causation also holds significant importance in start-ups. For instance, in contemporary business schools, topics like business plan formulation, competitive analysis, and business model design remain fundamental in the curriculum. These courses demonstrate goal-oriented rational decision-making thinking paradigms, notably focusing on causation.

A second implication of our findings concerns the relationship between causation and effectuation. As we have stated in our analysis of the existing literature, current research is inconsistent on this issue. Our results suggest a complementary relationship between causation and partial effectuation principles. Specifically, for start-ups, a configuration of causation and promotion-focused effectuation principles works best for improving firm performance. In comparison, for established firms, three configurations help them make better decisions: causation and promotion-focused effectuation principles, causation and prevention-focused effectuation principles, and causation and hybrid-focused effectuation principles.

Third, comparing solutions at different development stages allows us to consider possible high-performance development paths for firms as they mature. We offer three evolutionary paths for firms that must combine effectuation and causation to maintain stable performance. Path 1 can be called the continuation type (from causation and promotion-focused logic to causation and promotion-focused logic), which indicates that established firms can continue the decision-making logic of the start-up phase. Path 2 can be called the transformation type (from causation and promotion-focused logic to causation and prevention-focused logic), which shows that firms can significantly change how they make decisions as they grow. Path 3 can be called the switching type (from causation and promotion-focused logic to causation and hybrid-focused logic). There is no specific rule for this path—firms can increase or decrease certain decision-making principles according to their needs. This insight helps firms reflect on adjusting their decision-making methods to match better the use of causation and effectuation principles for firm growth.

### 5.2 Theoretical contributions

The study makes two significant contributions to the literature. First, we contribute to the ongoing debate on the relationship between causation and effectuation [2, 4–10]. While managers often rely on these two decision-making logics for critical strategic decision-making, insights are unclear as to how the two logics interplay with each other. We add new insights by distinguishing the different effects of each effectuation dimension and uncovering the dynamic relationships between the two logics.

Second, our study identifies three configurations of causation and effectuation and highlights their different roles during firms’ development stages. We provide new insights on how to mix and match causation and effectuation. As highlighted by Read et al. (2016, p. 531), “effectuation research needs to spell out in more detail […] useful ways to mix and match predictive and non-predictive strategies.” [18]. Specifically, our analysis reveals one approach for start-ups and three for established firms. While the precise configurations differ for start-ups and established firms, it is very notable that causation is a viable path to high performance.

### 5.3 Managerial implications

Our study offers compelling insights into management practice and management education. First, our findings reveal different configurations of causation and effectuation principles for high performance. This inspires managers to realize the complementary value between the two decision-making logics and encourages firms to be flexible in choosing specific logical combinations according to their situations [30]. Importantly, causation is the core element of each configuration. Firms may first set logic toward goal orientation and planning analysis and then combine certain effectuation principles to achieve high performance.

Second, the effectuation principles represent “teachable and learnable elements of entrepreneurial expertise” [36]. These effectuation principles have been extracted, and virtually “anyone can learn these tools” [18]. Our findings suggest it does not seem to pay to develop all effectuation principles. This inspires management education to focus more on effectuation principles and their differences than on effectuation as a single concept.

### 5.4 Limitations and future directions

There are also several limitations to this study. First, although this study examined the decision-making logic configuration of firms at different stages of development, we still need to provide complete evidence of changes in decision-making logic over time. Future research could use longitudinal data, conjoint experiment or machine learning methods to reveal more realistically the specific change processes of coexistence and alternation of firms’ decision-making logics over time [30–31, 76–80].

Second, the study only considered the contextual factor of the firm development stage, although many studies have shown that the firm development stage represents an important contextual factor [1, 2, 5, 31]. Causation and effectuation are widely perceived as the basic entrepreneurial decision-making logic to cope with external environmental uncertainty. Changes in the level and form of environmental uncertainty can affect the effects of entrepreneurial decision-making logic [9, 39, 81, 82]. Future research could further enrich our understanding of decision-making logic by incorporating environmental factors such as environmental uncertainty and formal institution [83–85].

## Supporting information

S1 Table Research on the relationship between causation, effectuation and firm performance. (PDF)

S2 Table Comparison between multiple regression analysis and configuration method. (PDF)

S3 Table Variable measurement items. (PDF)

S4 Table Interview company information. (PDF)

S1 File Questionnaire. (PDF)

S2 File Minimum data set. (XLSX)
