# Supplementary material for: Striking the balance: Configurations of causation and effectuation principles for SME performance
Source: PLoS One. 2024 Jun 28;19(6):e0302700. doi: 10.1371/journal.pone.0302700 (PMC11213296; doi:10.1371/journal.pone.0302700)
Supplement: S1 Table — (PDF) [file pone.0302700.s001.pdf]

S1 Table

Num	Author	Research question	Method	Main results
1	Brettel et al. (2012)	The study develops a multi-factor measurement model of effectuation and causation, and investigates their performance outcomes.	qualitative and quantitative analysis	(a) effectuation is positively related to success in highly innovative contexts, (b) causation approaches are beneficial in projects with low levels of innovativeness
2	Mckelvie et al. (2013)	What are the effects of effectuation and causation on firm-level performance in both the short and long run?	Regression analysis	Causation, flexibility, and the use of pre-commitments are dominant positive predictors of short-term relative performance. The use of effectuation and causation during firm emergence have a limited influence on the long-term performance of new ventures.
3	Mthanti and Urban (2014)	How do effectuation principles affect entrepreneurial orientation in the face of uncertainty in the external environment?	Regression analysis	Effectuation and its experimentation dimension are positively related to the level of entrepreneurial orientation in the firm. Effectuation acts as a moderator of the relationship between entrepreneurial orientation and firm performance.
4	Urban and Heydenryc (2015)	How are effectuation, technology orientation, and firm performance related?	Regression analysis	Effectuation and technology orientation are positively related. The dimensions of effectuation (pre-commitments, experimentation, and flexibility) significantly affect firm performance.
5	Roach et al. (2016)	How are effectuation and innovations related, and what is their impact on firm performance?	PLS structural equations	Two effectuation principles—available means and leverage contingencies—mediate the relationship between innovation orientation and product (service) innovations. The affordable loss principle is positively related to firm performance.
6	Guo et al.	How do the two venturing principles – effectuation	Regression analysis	Both effectuation and causation are positively associated

	(2016)	and causation – lead to venture growth?		with new internet venture growth. Effectuation leads to pioneering resource bundling, which in turn contributes to new internet venture growth. Causation also contributes to new internet venture growth, but through stabilizing resource bundling.
7	Parida et al. (2016)	How are effectuation and causation related to the probability of initial venture sales?	Regression analysis	The likelihood of initial sales increases when the entrepreneur places greater emphasis on causation and perceived control. Furthermore, the focus on perceived gains (as opposed to losses) strengthens the positive correlation between effectuation and initial sales.
8	Deligianni et al. (2017)	How does effectuation affect the association between product diversification and performance in new ventures?	Regression analysis	The effectuation dimensions of experimentation, flexibility, and pre-commitments positively moderate the association between product diversification and new venture performance.
9	Eijdenberg et al. (2017)	To what extent is small business growth determined by the influence of a founder's causal and effectual logic in conditions of uncertainty?	Regression analysis	Effectuation and causation do not have a significant impact on small businesses' growth in the context of uncertainty
10	Yu et al. (2018)	What is the impact of effectuation and causation, both together and individually, on firm performance in emerging markets?	Regression analysis	The results indicate that entrepreneurial businesses in emerging economies employ a combination of causation and effectuation strategies in highly unpredictable conditions, while preferring causation in less uncertain conditions.
11	Smolka et al. (2018)	Do effectuation and causation, jointly and separately, improve venture performance?	Regression analysis	Effectuation and causation positively influence the performance of ventures. The integration of effectuation and causation has a greater statistically significant positive impact on performance compared to each logic individually.
12	Palmié et al.	Do effectuation principles exert opposite effects on	Regression analysis	Promotion-focused (prevention-focused) principles are

	(2019)	the same criterion, and why?		positively (negatively) associated with a firm's EO. Some effectuation principles are more similar to causation in their underlying regulatory focus and their relationship with EO than they are to other effectuation principles.
13	An et al. (2020)	Which configurations of effectuation, causation, and bricolage are associated with high firm performance?	fsQCA method	Using a sample of 305 Chinese firms, the study finds six solutions explaining entrepreneurial processes in high-performing firms.
14	Shirokova et al. (2021)	How do effectuation and causation affect firm performance and performance variability in the context of an emerging market? and How does the economic crisis shape these relationships?	Regression analysis	Specifically, we show that for the companies impacted by negative circumstances, causation results in slight enhancements in performance but with a high degree of variability, whereas effectuation leads to performance increases along with increased predictability.
15	Harms et al. (2021)	Which configurations of causation and effectuation are associated with business model innovation?	fsQCA method	A second contingency is a partnership, and there is a trade-off between networking and affordable loss.
16	Deligianni et al. (2022)	How do new ventures faced with important resource constraints and high degrees of uncertainty manage to innovate?	Regression analysis	Experimentation, affordable loss, and flexibility (but not pre-commitments) are positively related to new venture innovation.
17	Taghvaee and Talebi (2022)	Should the facilitating role of market uncertainty on the MO–new product performance relationship be stronger when firms adopt effectuation?	Regression analysis	The dimensions of effectuation (except for affordable loss orientation) strengthen the MO–new product performance relationship in uncertain markets.
18	Zhang et al. (2023)	The purpose of this paper is to explore the relationship between effectuation, causation and firm performance.	Meta-analysis	The results show a positive correlation between two decision-making logics and firm performance and the influence of effectuation in firm performance is slightly stronger. However, the application environment is different: in the emerging market, the causation is more effective.
