# Supplementary material for: Striking the balance: Configurations of causation and effectuation principles for SME performance
Source: PLoS One. 2024 Jun 28;19(6):e0302700. doi: 10.1371/journal.pone.0302700 (PMC11213296; doi:10.1371/journal.pone.0302700)
Supplement: S2 Table — (PDF) [file pone.0302700.s002.pdf]

S2 Table

Categories	Regression analysis method	Configuration method
Theoretical objective	Testing and refining theory	Testing, refining and constructing theory
Research question	Net effect issue	Configuration issue
Causality assumption	Causal monotonicity (constancy, consistency and symmetry)	Causal complexity (equifinality, asymmetry)
Study sample	Large sample	Not required
Logical reasoning	Deductive reasoning	Retrospective reasoning
Mathematical foundation	Analytics	Set theory
