# Supplementary material for: Striking the balance: Configurations of causation and effectuation principles for SME performance
Source: PLoS One. 2024 Jun 28;19(6):e0302700. doi: 10.1371/journal.pone.0302700 (PMC11213296; doi:10.1371/journal.pone.0302700)
Supplement: S3 Table — (PDF) [file pone.0302700.s003.pdf]

S3 Table

Variable	Brief items
**Causation**<br>Chandler et al. (2011)	1.Analyze long run opportunities and select what will provide the best returns.<br>2.Develop a strategy to best take advantage of resources and capabilities.<br>3.Design and plan business strategies.<br>4.Organize and implement control processes to make sure we meet objectives.<br>5.Research and select target markets and do meaningful competitive analysis.<br>6.Have a clear and consistent vision for where we want to end up.<br>7.Design and plan production and marketing efforts.
**Experimentation**<br>Chandler et al. (2011)	1.We experimented with different products and/or business models.<br>2.Our current product/service offerings are very different from what was originally envisioned.<br>3.We tried a number of different approaches until we found a business model that worked.
**Flexibility**<br>Chandler et al. (2011)	1.We allowed the business to evolve as opportunities emerged.<br>2.We adapted what we were doing to the resources we had.<br>3.We were flexible and took advantage of opportunities as they arose.<br>4.We avoided courses of action that restricted our flexibility and adaptability.
**Affordable loss**<br>Chandler et al. (2011)	1.We were careful not to commit more resources than we could afford to lose.<br>2.We were careful not to risk more money than we were willing to lose with our initial idea.<br>3.We were careful not to risk so much money that the company would be in real trouble financially if things didn't work out.
**Pre-commitments**<br>Chandler et al. (2011)	1.We used a substantial number of agreements with customers, suppliers and other organizations and people to reduce the amount of uncertainty.<br>2.We used pre-commitments from customers and suppliers as often as possible.<br>3.Social networks provide us with low-cost resources.<br>4.Working with outsiders and companies has greatly enhanced our capabilities.<br>5.We have been working on developing alliances with other individuals and organizations.<br>6.Our partnerships with other organizations and individuals play a key role in our ability to provide products and services.
**Firm performance**<br>Dess and Robinson (1984)	1.What is your firm's performance relative to other software firms in your city?<br>2.What is your firm's performance relative to other software firms in your market?<br>3.What is your firm's performance relative to your competitors in your industry?
