# Supplementary material for: Striking the balance: Configurations of causation and effectuation principles for SME performance
Source: PLoS One. 2024 Jun 28;19(6):e0302700. doi: 10.1371/journal.pone.0302700 (PMC11213296; doi:10.1371/journal.pone.0302700)
Supplement: S4 Table — (PDF) [file pone.0302700.s004.pdf]

S4 Table

	Company A	Company B
Main business	Company A is a food manufacturing company that mainly produces hot sauce products. It mainly focuses on takeout market.	Company B focuses on the manufacturing, sales and application integration of industrial automation products, and is committed to providing core components and system integration solutions for smart factories.
Firm age	10	13
Number of employees	141	240
Location	Hangzhou	Shanghai
Duration of interviews	75 minutes	60 minutes
