# Supplementary material for: Striking the balance: Configurations of causation and effectuation principles for SME performance
Source: PLoS One. 2024 Jun 28;19(6):e0302700. doi: 10.1371/journal.pone.0302700 (PMC11213296; doi:10.1371/journal.pone.0302700)
Supplement: S1 File — (PDF) [file pone.0302700.s005.pdf]

S1 File

Please introduce your basic information.

**Demographic Data:**	
Name	
Gender	☐Male ☐Female
Age	☐29 years or younger ☐30-40 years ☐41-50 years ☐51 years or older
Education	☐Lower than bachelor ☐Bachelor ☐Master ☐Ph.D.

Please introduce the basic information of your company.

**Firm Information:**	
Firm name	
Number of employees	
Year Established	
Location	☐Beijing ☐Hangzhou ☐Shanghai ☐Changchun
Ownership	☐Private enterprise ☐Joint enterprise ☐State ownership ☐Foreign enterprise

The following questions examine the logical approach you prefer to use when making strategic decisions. Please evaluate the extent to agree with the following descriptions. Please use the five-point scale as shown.

**Causation**	
1. Analyze long run opportunities and select what will provide the best returns.	1 2 3 4 5
2. Develop a strategy to best take advantage of resources and capabilities.	1 2 3 4 5
3. Design and plan business strategies.	1 2 3 4 5
4. Organize and implement control processes to make sure we meet objectives.	1 2 3 4 5
5. Research and select target markets and do meaningful competitive analysis.	1 2 3 4 5
6. Have a clear and consistent vision for where we want to end up.	1 2 3 4 5
7. Design and plan production and marketing efforts.	1 2 3 4 5
**Experimentation**	
1. We experimented with different products and/or business models.	1 2 3 4 5
2. Our current product/service offerings are very different from what was originally envisioned.	1 2 3 4 5
3. We tried a number of different approaches until we found a business model that worked.	1 2 3 4 5
**Flexibility**	
1. We allowed the business to evolve as opportunities emerged.	1 2 3 4 5
2. We adapted what we were doing to the resources we had.	1 2 3 4 5
3. We were flexible and took advantage of opportunities as they arose.	1 2 3 4 5
4. We avoided courses of action that restricted our flexibility and adaptability.	1 2 3 4 5
**Affordable loss**	1 2 3 4 5
1. We were careful not to commit more resources than we could afford to lose.	1 2 3 4 5
2. We were careful not to risk more money than we were willing to lose with our initial idea.	1 2 3 4 5
3.We were careful not to risk so much money that the company would be in real trouble	1 2 3 4 5

financially if things didn't work out.	
**Pre-commitments**	
1. We used a substantial number of agreements with customers, suppliers and other organizations and people to reduce the amount of uncertainty.	1 2 3 4 5
2. We used pre-commitments from customers and suppliers as often as possible.	1 2 3 4 5
3. Social networks provide us with low-cost resources.	1 2 3 4 5
4. Working with outsiders and companies has greatly enhanced our capabilities.	1 2 3 4 5
5. We have been working on developing alliances with other individuals and organizations.	1 2 3 4 5
6. Our partnerships with other organizations and individuals play a key role in our ability to provide products and services.	1 2 3 4 5

Please evaluate the extent to agree with the following descriptions. Please use the seven-point scale as shown.

**Firm performance**	
1. What is your firm's performance relative to other software firms in your city?	1 2 3 4 5 6 7
2. What is your firm's performance relative to other software firms in your market?	1 2 3 4 5 6 7
3. What is your firm's performance relative to your competitors in your industry?	1 2 3 4 5 6 7
